# Topographic comparison of the retinal microvascular changes between patients with compressive and glaucomatous optic neuropathies

**DOI:** 10.1038/s41598-023-50068-6

**Published:** 2023-12-19

**Authors:** Hyunah Lim, Byung Joo Lee, Michael S. Kook, Kyung Rim Sung, Ko Eun Kim, Yeji Moon

**Affiliations:** Department of Ophthalmology, Asan Medical Center, University of Ulsan College of Medicine, 88, Olympic-ro 43-gil, Songpa-gu, Seoul, 05505 Republic of Korea

**Keywords:** Neuro-vascular interactions, Visual system

## Abstract

We investigated the difference in optical coherence tomography angiography characteristics between the patients with compressive optic neuropathy (CON, n = 26) and glaucomatous optic neuropathy (GON, n = 26), who were matched for the severity of visual field defect. The peripapillary retinal nerve fiber layer (pRNFL) thickness in the nasal and temporal sectors was thinner in the CON group, whereas the inferior pRNFL thickness was thinner in the GON group. Accordingly, the CON group had lower peripapillary vessel density (pVD) in the nasal and temporal sectors, and the GON group in the inferior sector. In the macular area, the CON group had a thinner macular ganglion cell-inner plexiform layer in the superior and nasal sectors, whereas the GON group in the inferior sector. However, the CON group did not have a lower macular VD than the GON group in any sector, whereas the GON group exhibited lower superficial capillary plexus VD in the superior, inferior, and temporal sectors. Comparison of the structure–vasculature correlation revealed a significant difference in the nasal and temporal peripapillary areas and superior and nasal macular sectors; a decrease in VD was greater in the GON group than in the CON group when the comparable structural change occurred.

## Introduction

Increased intraocular pressure (IOP) is the most important risk factor for glaucomatous optic neuropathies (GON), and IOP reduction can slow the progression rate of visual loss caused by GON including normal tension glaucoma (NTG)^1–4^. Although the pathogenesis of GON is not fully understood, IOP can cause mechanical stress and strain on the lamina cribrosa and adjacent tissues, inducing axonal damage and disruption of axonal transport^5–7^. Similarly, compressive optic neuropathy (CON) is caused by a mechanical compression of the optic nerve; the optic chiasm, in particular, is at risk of compression by several intracranial tumors, namely pituitary adenoma, Rathke cleft cyst, craniopharyngioma, and parasellar meningioma.

Despite similarities in the etiology of mechanical pressure, CON and GON differ in their clinical characteristics. Several studies have compared the structural changes between CON and GON^8–12^, focusing on the different topographical patterns of structural damage on the optic disc, peripapillary retinal nerve fiber layer (pRNFL), and macular ganglion cell-inner plexiform layer (mGCIPL). Lei et al. recently reported the difference in microvasculature between CON and GON^13^. However, they included CON with glaucoma-like cupping, which were not representative of typical CON with optic chiasm compression. Comparing the microvasculature of CON and GON would help clinicians understand their pathophysiology and differentiate the two diseases, given that non-IOP-dependent mechanisms, such as vascular insufficiency, may induce optic nerve damage in GON^14,15^. Therefore, this study aimed to investigate the peripapillary and macular microvasculature in patients with CON and GON and identify differences in both diseases, using optical coherence tomography angiography (OCTA).

## Results

A total of 78 eyes, 26 each in the CON, GON, and normal control (NC) groups, were included in the final analysis (Table 1). No significant difference in the mean deviation (MD) of the visual field (VF) test was observed between the CON and GON groups; however, the best-corrected visual acuity (BCVA) was worse in the CON group than in the GON group (0.30 ± 0.49 vs. 0.04 ± 0.08 logMAR, *p* = 0.002).

**Table 1:** Baseline demographic data of the study population.

	Compressive optic neuropathy (CON, n = 26)	Glaucomatous optic neuropathy (GON, n = 26)	Normal control (NC, n = 26)	*p* value		
				CON vs. NC	GON vs. NC	CON vs. GON
Age, years	55.7 ± 13.8	54.8 ± 12.5	55.7 ± 12.1	0.949	0.754	0.867
Sex, female, n (%)*	7 (26.9)	7 (26.9)	12 (46.2)	0.249	0.249	> 0.999
Best corrected visual acuity, logMAR	0.30 ± 0.49	0.04 ± 0.08	0.01 ± 0.03	** < 0.001**	0.138	**0.002**
Intraocular pressure, mmHg	15.8 ± 2.9	14.7 ± 3.6	14.4 ± 2.9	0.065	0.844	0.091
Refractive errors, D	− 1.0 ± 2.9	− 1.5 ± 3.0	− 0.6 ± 1.8	0.706	0.227	0.464
Mean deviation, dB	− 9.4 ± 7.5	− 9.8 ± 7.5	0.3 ± 1.1	** < 0.001**	** < 0.001**	0.796
Intracranial tumor*		N/A	N/A			
Pituitary adenoma	19 (73.1)					
Craniopharyngioma	3 (11.5)					
Parasellar meningioma	2 (7.7)					
Rathke cleft cyst	2 (7.7)					

Significant values are in bold.

*logMAR* the logarithm of minimum angle of resolution, *D* diopter.

*Values are presented as the number of patients (%).

### Comparison of structural and vascular changes in peripapillary area

Table 2 depicts the peripapillary structural and vascular changes of the three groups. The average pRNFL thickness was 71.5 ± 10.0 μm in the CON group and 72.5 ± 10.7 μm in the GON group. There was no significant difference in the average pRNFL thickness between two disease groups (*p* = 0.733), however both groups had a thinner pRNFL thickness compared to the NC group. The pRNFL thickness in the nasal and temporal quadrant was thinner in the CON group than in the GON and NC groups, whereas the inferior pRNFL thickness was thinner in the GON group than in the CON and NC groups. Accordingly, the peripapillary vessel density (pVD) in the nasal and temporal quadrant was lower in the CON group than in the GON and NC groups, whereas the pVD in the inferior sector was lower in the GON group than in the CON and NC groups.

**Table 2:** Comparison of structural and vascular changes of peripapillary area between groups.

	Compressive optic neuropathy (CON)	Glaucomatous optic neuropathy (GON)	Normal control (NC)	*p* value			Comparison
				CON vs. NC	GON vs. NC	CON vs. GON	
pRNFL thickness, *μ*m							
Average	71.5 ± 10.0	72.5 ± 10.7	98.0 ± 6.7	** < 0.001**	** < 0.001**	0.733	**C = G < N**
Superior (S)	90.7 ± 16.0	90.1 ± 22.6	123.2 ± 12.4	** < 0.001**	** < 0.001**	0.589	**C = G < N**
Nasal (N)	56.6 ± 5.1	66.7 ± 7.4	68.0 ± 8.0	** < 0.001**	0.515	** < 0.001**	**C < G = N**
Inferior (I)	91.5 ± 17.5	74.9 ± 17.4	128.2 ± 13.4	** < 0.001**	** < 0.001**	** < 0.001**	**G < C < N**
Temporal (T)	47.4 ± 11.3	57.2 ± 13.7	70.7 ± 10.5	** < 0.001**	** < 0.001**	**0.009**	**C < G < N**
Peripapillary vessel density (%)							
Superior (S)	41.3 ± 8.9	41.1 ± 11.1	53.6 ± 3.1	** < 0.001**	** < 0.001**	0.960	**C = G < N**
Nasal (N)	33.2 ± 6.7	41.5 ± 7.2	49.3 ± 2.4	** < 0.001**	** < 0.001**	** < 0.001**	**C < G < N**
Inferior (I)	42.3 ± 7.4	33.3 ± 9.1	54.3 ± 2.6	** < 0.001**	** < 0.001**	** < 0.001**	**G < C < N**
Temporal (T)	40.3 ± 8.3	47.3 ± 10.3	56.0 ± 2.7	** < 0.001**	** < 0.001**	**0.004**	**C < G < N**

Significant values are in bold.

*pRNFL* peripapillary retinal nerve fiber layer, *C* compressive optic neuropathy, *G* glaucomatous optic neuropathy, *N* normal control.

### Comparison of structural and vascular changes in the macular area

Compared with the NC group, the mGCIPL thickness in all sectors decreased in the CON and GON groups (Table 3). The average mGCIPL thickness was 63.0 ± 7.3 μm in the CON group and 66.4 ± 9.1 μm in the GON group (*p* = 0.119). Comparison of sectoral mGCIPL thickness between the two disease groups revealed the CON group had a thinner mGCIPL in the superior and nasal sectors, whereas the GON group exhibited a thinner mGCIPL in the temporal sector.

**Table 3:** Comparison of structural and vascular changes of macular area between groups.

	Compressive optic neuropathy (CON)	Glaucomatous optic neuropathy (GON)	Normal control (NC)	*p* value			Comparison
				CON vs. NC	GON vs. NC	CON vs. GON	
mGCIPL thickness, *μ*m							
Average	62.7 ± 6.8	66.4 ± 9.1	83.0 ± 4.3	** < 0.001**	** < 0.001**	0.083	**C = G < N**
Superior (S)	60.3 ± 8.7	70.0 ± 11.9	84.0 ± 5.2	** < 0.001**	** < 0.001**	**0.004**	**C < G < N**
Nasal (N)	55.1 ± 8.2	71.0 ± 11.1	84.2 ± 4.9	** < 0.001**	** < 0.001**	** < 0.001**	**C < G < N**
Inferior (I)	63.0 ± 7.7	60.7 ± 9.8	80.7 ± 5.3	** < 0.001**	** < 0.001**	0.249	**C = G < N**
Temporal (T)	72.2 ± 9.5	62.2 ± 8.9	82.5 ± 4.5	** < 0.001**	** < 0.001**	** < 0.001**	**G < C < N**
Macular vessel density, SCP (%)							
Superior (S)	46.0 ± 4.9	42.7 ± 6.0	52.9 ± 3.4	** < 0.001**	** < 0.001**	**0.047**	**G < C < N**
Nasal (N)	45.3 ± 4.9	44.7 ± 6.0	53.3 ± 3.1	** < 0.001**	** < 0.001**	0.775	**C = G < N**
Inferior (I)	45.7 ± 4.9	40.0 ± 5.8	51.7 ± 3.7	** < 0.001**	** < 0.001**	** < 0.001**	**G < C < N**
Temporal (T)	45.7 ± 3.7	39.9 ± 5.8	49.8 ± 3.6	** < 0.001**	** < 0.001**	** < 0.001**	**G < C < N**
Macular vessel density, DCP (%)							
Superior (S)	49.0 ± 7.0	46.2 ± 7.5	53.2 ± 5.7	**0.044**	** < 0.001**	0.164	**C = G < N**
Nasal (N)	50.8 ± 5.5	47.6 ± 8.2	53.6 ± 5.5	0.081	**0.002**	0.119	**G < N**
Inferior (I)	48.4 ± 7.0	42.5 ± 9.0	51.8 ± 6.5	0.081	** < 0.001**	**0.022**	**G < C = N**
Temporal (T)	52.6 ± 5.4	47.0 ± 7.3	54.9 ± 5.1	0.128	** < 0.001**	**0.007**	**G < C = N**

Significant values are in bold.

*mGCIPL* macular ganglion cell-inner plexiform layer, *SCP* superficial capillary plexus, *DCP* deep capillary plexus, *C* compressive optic neuropathy, *G* glaucomatous optic neuropathy, *N* normal control.

Both groups also exhibited lower macular superficial capillary plexus (SCP) vessel density (VD) across all areas compared with the NC group. However, the CON group did not exhibit lower SCP VD than the GON group in any sector. Comparatively, the GON group exhibited lower SCP VD in the superior, inferior, and temporal sectors. Comparing the macular deep capillary plexus (DCP) VD showed that the CON group had a significant decrease only in the superior sectors compared with the NC group. However, the DCP VD decreased in all sectors in the GON group compared with the NC group. In addition, the GON group had lower DCP VD than the CON group in the inferior and temporal sectors.

### Correlation between structural and vascular changes

We topographically investigated the correlation between structural and vascular change in the CON and GON groups (Table 4). Both groups showed significant correlations between structural and vascular changes in the peripapillary area. Only in the nasal sector the CON group had correlation with borderline significance (*p* = 0.057). Similarly, in the macular area, both groups showed significant correlation between mGCIPL thickness and the SCP VD. However, both groups had no significant correlation between mGCIPL thickness and the DCP VD.

**Table 4:** The correlation between structural and vascular changes in peripapillary and macular area between groups.

	Compressive optic neuropathy (CON)		Glaucomatous optic neuropathy (GON)	
	Rho	*p* value	Rho	*p* value
pRNFL thickness and peripapillary VD				
Superior (S)	0.783	** < 0.001**	0.754	** < 0.001**
Nasal (N)	0.317	0.057	0.348	**0.041**
Inferior (I)	0.801	** < 0.001**	0.799	** < 0.001**
Temporal (T)	0.503	**0.004**	0.436	**0.013**
mGCIPL thickness and SCP VD				
Superior (S)	0.649	** < 0.001**	0.425	**0.015**
Nasal (N)	0.509	**0.004**	0.485	**0.006**
Inferior (I)	0.465	**0.008**	0.693	** < 0.001**
Temporal (T)	0.496	**0.005**	0.641	** < 0.001**
mGCIPL thickness and DCP VD				
Superior (S)	− 0.009	0.483	0.220	0.140
Nasal (N)	− 0.256	0.103	0.100	0.314
Inferior (I)	− 0.170	0.204	0.092	0.327
Temporal (T)	0.018	0.464	− 0.036	0.430

Significant values are in bold.

*pRNFL* peripapillary retinal nerve fiber layer, *VD* vessel density, *mGCIPL* macular ganglion cell-inner plexiform layer, *SCP* superficial capillary plexus, *DCP* deep capillary plexus.

**Figure 1:**
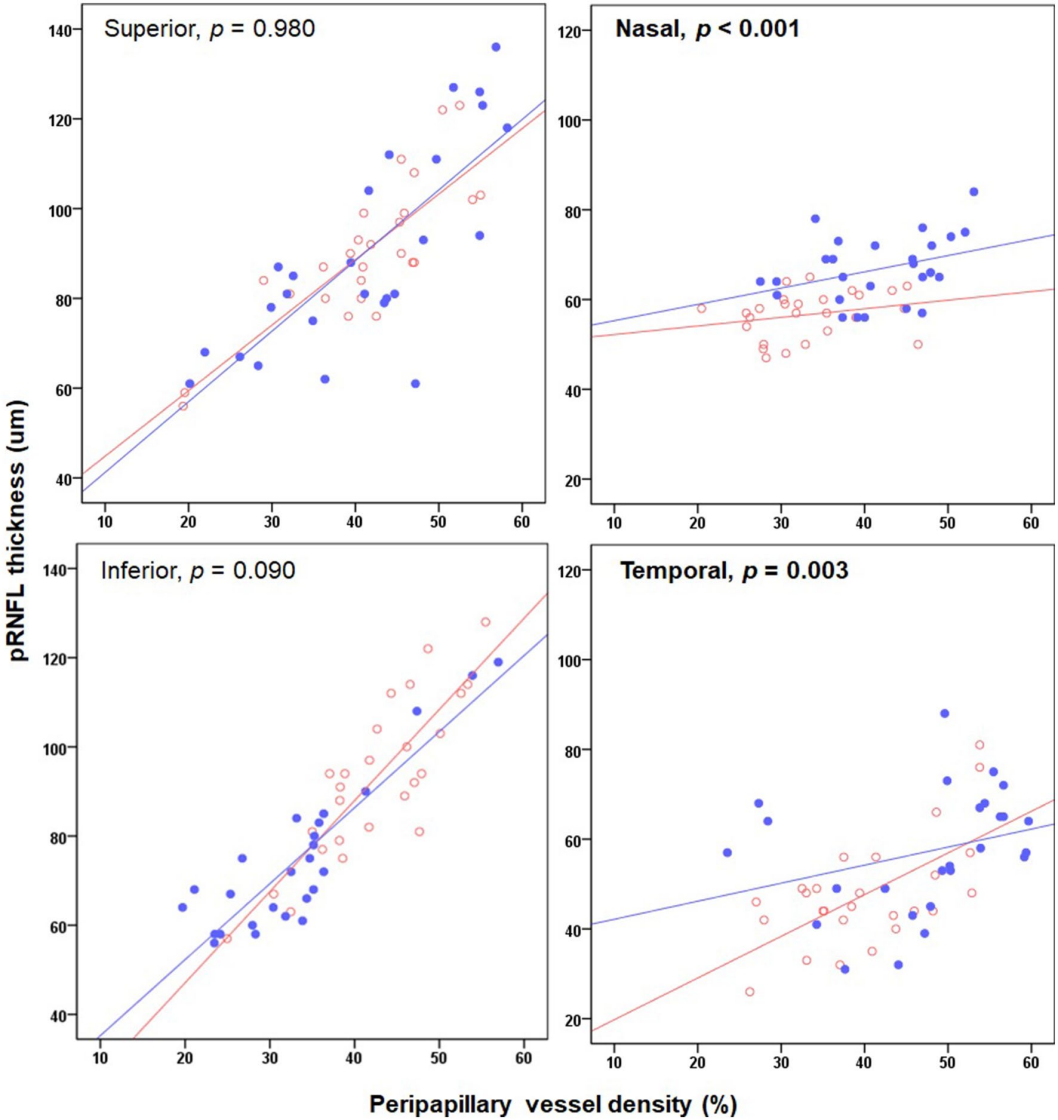
Scatter plot showing the correlation between structural and vascular changes in the peripapillary area in the CON and GON groups. Red open dots indicate the cases of the CON group, and blue closed dots indicate the case of the GON group.

**Figure 2:**
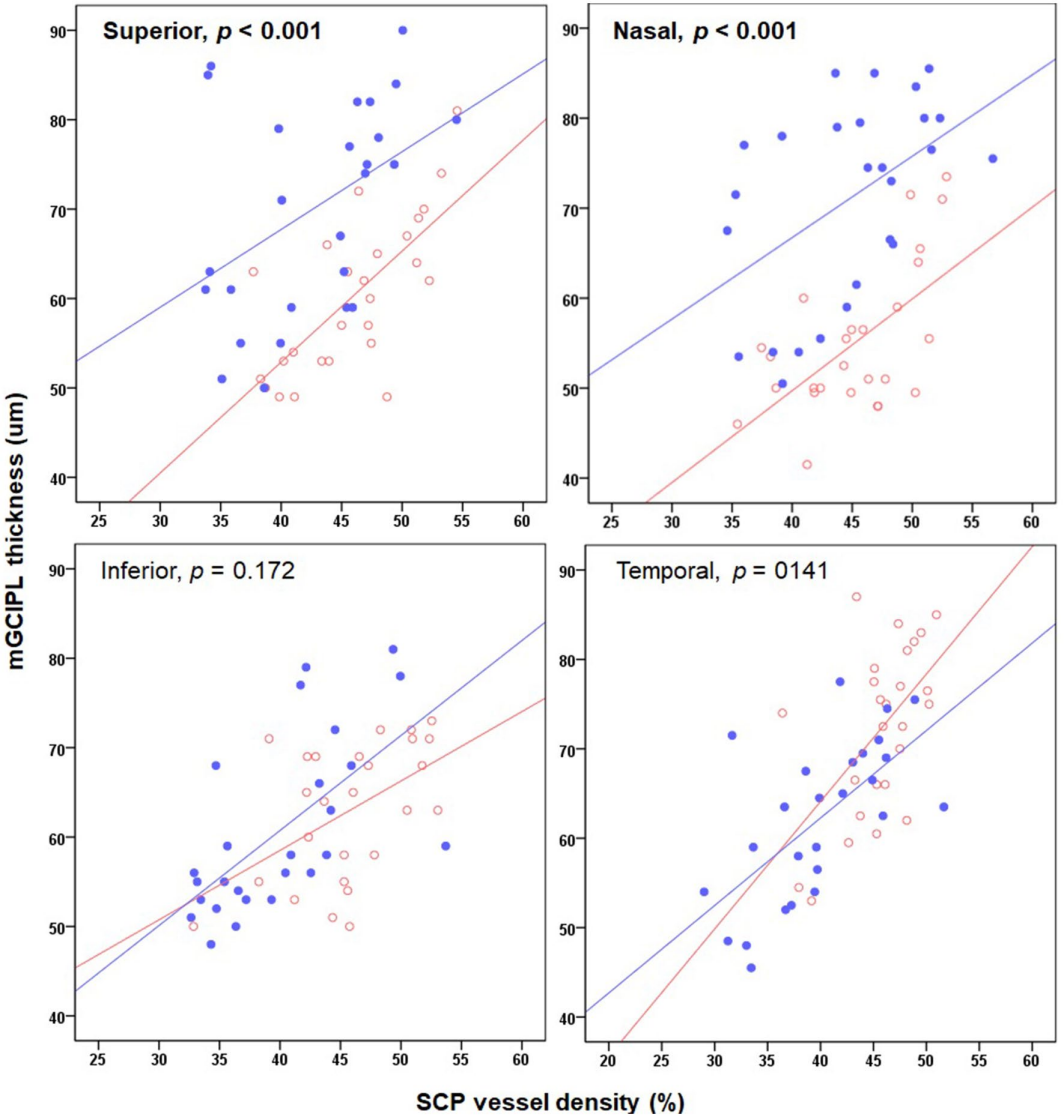
Scatter plot showing the correlation between structural and vascular changes in the macular area in the CON and GON groups. Red open dots indicate the cases of the CON group, and blue closed dots indicate the case of the GON group.

Lastly, we determined the difference in the structure–vasculature correlation between the two disease groups. In the nasal and temporal peripapillary area, there was a significant difference in the structure–vascular correlation between the two groups (*p* < 0.001 and *p* = 0.003, respectively) (Fig. 1). In addition, the correlation between the SCP VD and mGCIPL thickness differed between the two groups in the superior and nasal macular sectors (all *p* < 0.001) (Fig. 2). In these four areas, the trend line of the CON group is located inferior to that of the GON group, indicating that a decrease of VD was greater in the GON group than in the CON group when comparable structural changes occurred.

## Discussion

In this OCTA study, we compared the vascular changes between patients with GON and CON. Both groups showed decreased pVD, particularly in the area where pRNFL thinning was prominent. Compression on the optic chiasm due to parasellar mass initially affects the crossing fibers, which are axons originating from the nasal hemiretina. These fibers pass through the temporal and nasal sectors of the optic nerve head, resulting in pRNFL thinning predominantly in these sectors^9,16–18^. Meanwhile, arcuate fibers entering the superior and inferior optic nerve head are more susceptible to glaucomatous damage because of the larger size of the pores of the lamina cribrosa with lesser connective tissue support^5^. Thus, as expected, pRNFL thinning was remarkable in the nasal and temporal quadrants in the CON group and the inferior quadrant in the GON group in our study. Accordingly, the patients with CON had lower pVD in the temporal and nasal sectors of the peripapillary area, while those with GON in the inferotemporal sector.

In addition to the topographical agreement between the areas with reduced pVD and those with reduced pRNFL thickness, the quantitative correlation between pVD and pRNFL thickness was revealed in this study. This finding may be the result of intraocular microvascular changes secondary to optic atrophy, as reported in the literature^19–21^. Damage to the axons can reduce metabolic activity and affect the nutrient demand and blood supply, leading to changes in the corresponding vasculature^22^.

However, in the CON group, topographical change of mGCIPL thickness did not completely correspond to that of macular VD. Although the macular SCP VD in the patients with CON decreased compared with that of the healthy participants, as in a previous study^21^, the SCP VD was not significantly lower in the CON group than in the GON group, even in the sectors where mGCIPL thinning was more prominent. In contrast, the GON group showed a significant decrease of the SCP VD in the superior, inferior, and temporal macular areas compared with the CON group, indicating that the area in which the SCP VD was lower in the GON group than in the CON group was larger than the area where mGCIPL thickness was thinner than that of the CON group. In addition, the GON group showed a greater change in the macular DCP VD; the GON group had a significant decrease in macular DCP VD in all areas compared with the NC group and in half of the areas compared with the CON group. The difference in the DCP VD between the two optic neuropathies is inconsistent with the previous study^13^. As mentioned above, Lei et al. compared the microvasculature between CON with glaucoma-like cupping and GON and reported no significant difference in DCP VD between the two optic neuropathies. In this study, we included patients with typical CON who had undergone intracranial surgeries to optic chiasm decompression more than 6 months prior. This would have affected the results in comparing the DCP VD, and our findings suggest the microvascular changes are dominant in the GON group.

Moreover, the comparison of the structure–vasculature correlation between the two disease groups indicates that the decrease in VD was smaller in the CON group, even in the area where the structural change was more prominent compared with the GON group. This discrepancy in the severity of vascular changes between the two groups suggests that vascular changes may play different roles in the two optic neuropathies. Several previous studies have reported that OCTA changes may precede pRNFL changes in glaucoma, which support the “vascular theory,” postulating retinal ganglion cell death as a consequence of reduced blood supply^23,24^. It should be noted that the GON group included the patients with NTG (18 eyes, 69.2%) whose maximum IOP was less than 21 mmHg. Although reduction in IOP has a significant effect on the progression of NTG, vascular dysfunction has been considered an important factor in the progression of NTG^25,26^. The high proportion of NTG in this study may also have contributed to the results.

We also wish to highlight the difference in the location of lesions between CON and GON. Lamina cribrosa strain arising from the interactions of multiple forces plays a key role in GON^27,28^. The lamina cribrosa is only 5 mm from the fovea and contains capillaries; consequently, the mechanical strain may directly affect the retinal blood flow^29^. Meanwhile, parasellar mass applies pressure to the optic chiasm, which is 50 mm from the optic disc, and cannot directly disturb the blood supply to the retina.

This study has limitations as a cross-sectional study with a relatively small sample size. It is not a longitudinal study, and therefore cannot determine the temporal relationship between vascular, structural, and functional changes in the retina. Although the sectors of the OCT and OCTA images were not consistent, the difference in vascular changes can be clarified by comparing those in each topographical area between two optic neuropathies with comparable functional defects. Moreover, these findings suggest that the vascular changes in GON might be the primary lesion, as well as the secondary changes following damage to retinal ganglion cells and their axons.

In conclusion, vascular and structural changes corresponded well in both optic neuropathies. However, the CON group exhibited relatively preserved vasculature, while the GON group exhibited a decrease in microvasculature in a larger area and with greater severity. Our results can help physicians elucidate the pathophysiology of both optic neuropathies.

## Methods

This retrospective cross-sectional study was approved by the institutional review board of Asan Medical Center. The study procedures were conducted in accordance with the tenets of the Declaration of Helsinki. The requirement for informed consent was waived due to the retrospective study design by the institutional review board of Asan Medical Center.

### Participants

We reviewed the medical records of patients who visited the neuro-ophthalmologic clinic and were diagnosed with CON at Asan Medical Center from April 2019 to October 2020. All patients in the CON group had intracranial tumors compressing the optic chiasm, confirmed by magnetic resonance imaging (MRI). These patients also had VF defects at the time of tumor diagnosis. Considering the clinical course of visual improvement after surgery^30^, data obtained ≥ 6 months after intracranial surgery were used for the final analysis^8,9^, and the eye with the more severe VF defect was selected for the final analysis. Moreover, only the patients without recurrent tumors confirmed by MRI were included. Patients with CON with an IOP of > 21 mmHg or with definite glaucomatous optic nerve damage were excluded from this study.

Patients in the GON group were diagnosed with primary open-angle glaucoma by a glaucoma specialist at Asan Medical Center based on the glaucomatous optic nerve damage and associated VF defects. They had no other ocular disease that could be associated with increased IOP and had an open anterior chamber angle on gonioscopy. For the final analysis, the eyes with GON were selected by matching for age, refraction, and the severity of VF defect based on the MD of the Humphrey perimetry (Carl Zeiss Meditec, Dublin, CA, USA). To make the pattern of VF defect consistent within the group, as the CON group mainly showed temporal VF defects, the GON group in this study consisted of patients whose superior VF defect was larger than the inferior VF defect as assessed by a glaucoma specialist. Data from age- and refraction-matched NC were also obtained. All NC groups had an IOP of less than 21 mmHg and were confirmed free of ocular disease. More than two authors confirmed that each NC had normal VF test results and there was no test point with P < 1% on the PSD plot in the NC group. To exclude the effect of intra-individual factors, only one eye of one patient was included in the final analysis in the GON and NC groups.

All participants underwent a comprehensive ophthalmic examination, including measurements of BCVA, IOP by using Goldmann applanation tonometry, and refractive error by using an autorefractor (KR-890; Topcon Corp, Tokyo, Japan), as well as a slit-lamp biomicroscopy and fundus examination. An automated VF test was performed in patients with CON using the Humphrey perimetry SITA-Standard 30-2 program and in those with GON using the SITA-Standard 24-2 program.

### OCT/OCTA image acquisition and procession

**Figure 3:**
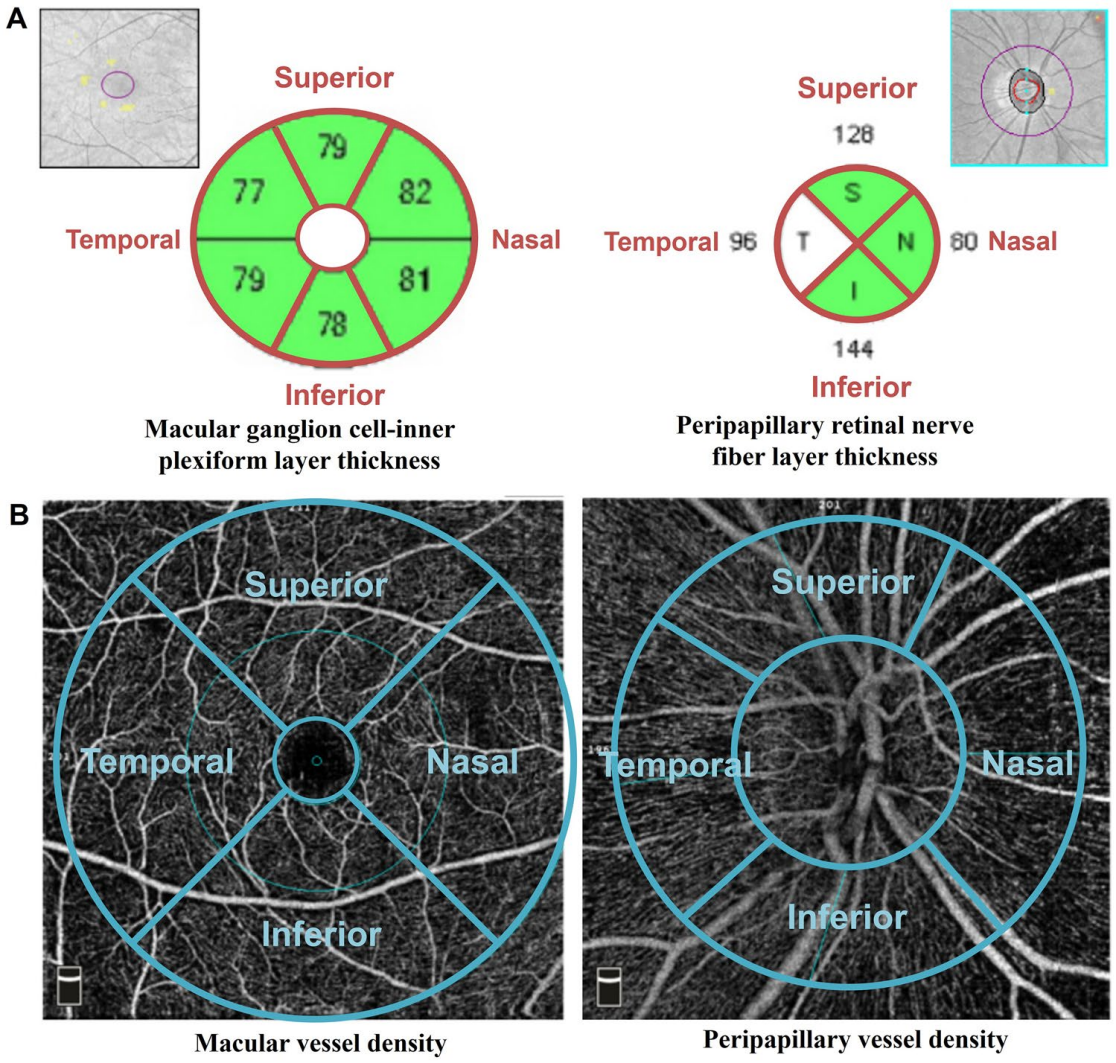
Sectorial division of (**A**) the OCT and (**B**) the OCTA image.

Cirrus OCT (Carl Zeiss Meditec, Dublin, CA, USA) software automatically tracked the geometric center of the optic disc and measured the pRNFL thickness along a 3.46 mm-diameter circumpapillary circle. The pRNFL thickness was measured in each of the four quadrants of the affected eye (temporal, superior, inferior, and nasal) around the disc. The mGCIPL thickness was measured in the elliptical annulus centered on the macula. The inner ring had a vertical diameter of 1 mm and a horizontal diameter of 1.2 mm, while the outer ring had a vertical diameter of 4 mm and a horizontal diameter of 4.8 mm. The average mGCIPL thickness and six sectoral (superotemporal, superior, superonasal, inferonasal, inferior, and inferotemporal) mGCIPL thicknesses were extracted. Temporal mGCIPL thickness was determined as the average value of the superotemporal and inferotemporal mGCIPL thicknesses, and the nasal mGCIPL thickness as the average value of the superonasal and inferonasal mGCIPL thicknesses. Finally, we used mGCIPL thicknesses of four macular sectors (Fig. 3A).

All participants were examined using the AngioVue OCTA imaging system (Optovue Inc, Fremont, CA, USA): a 6 × 6 mm^2^ macular cube scan centered automatically on the fovea and a 4.5 × 4.5 mm^2^ cube scan centered automatically on the optic disc were performed (Fig. 3B). Eyes with a poor image quality with a signal strength index < 45, aberrant segmentation, or movement-related artifacts were excluded.

The peripapillary VD (pVD) was measured within a 750 μm-wide annulus extending from the optic disc boundary automatically segmented in the radial peripapillary capillary. The average pVD of eight sectoral peripapillary areas (temporosuperior, superotemporal, superonasal, nasosuperior, nasoinferior, inferonasal, inferotemporal, and temporoinferior) was measured. Then, the VD of the superior sector was obtained by averaging the VD of the superotemporal and superonasal sectors. In the same way, the VD of the nasal, inferior, and temporal sectors was obtained.

The macular parameters in each of the four quadrants (temporal, superior, nasal, and inferior) were measured as follows: (1) the parafoveal VD within an annulus centered at the fovea with an inner diameter of 1.0 mm and an outer diameter of 3.0 mm, and (2) the perifoveal VD in within an annulus centered at the fovea with an inner diameter of 3.0 mm and an outer diameter of 6.0 mm. The average value of the parafoveal and perifoveal VD were used in each quadrant. All macular parameters were measured in automatically segmented superficial capillary plexus (SCP) and deep capillary plexus (DCP) layers.

### Statistical analysis

The Mann–Whitney U and Fisher’s exact tests were performed to compare data sets between the two groups, as appropriate. Spearman’s rank correlation analysis was used to determine the correlation between structural and vascular changes. To identify the difference in the structure–vasculature correlation between the CON and GON groups, we performed ANCOVA (analysis of covariance) using the generalized linear model. *P* values of < 0.05 were considered statistically significant. All statistical analyses were performed using SPSS version 23.0 (IBM Corp., Armonk, NY, USA).

## Data Availability

The datasets generated and/or analyzed during the current study are available from the corresponding author on reasonable request.
